# Advances in anti-tumor based on various anaerobic bacteria and their derivatives as drug vehicles

**DOI:** 10.3389/fbioe.2023.1286502

**Published:** 2023-10-03

**Authors:** Daichen Song, Xiaofan Yang, Yanfei Chen, Pingping Hu, Yingying Zhang, Yan Zhang, Ning Liang, Jian Xie, Lili Qiao, Guodong Deng, Fangjie Chen, Jiandong Zhang

**Affiliations:** ^1^ Shandong Key Laboratory of Rheumatic Disease and Translational Medicine, Department of Oncology, Shandong Lung Cancer Institute, The First Affiliated Hospital of Shandong First Medical University and Shandong Provincial Qianfoshan Hospital, Jinan, China; ^2^ School of Clinical Medicine, Jining Medical University, Jining, China

**Keywords:** bacteria, bacteria-derived membrane vesicles, drug delivery systems, cancer therapy, outer membranes vesicles

## Abstract

Cancer therapies, such as chemotherapy and radiotherapy, are often unsatisfactory due to several limitations, including drug resistance, inability to cross biological barriers, and toxic side effects on the body. These drawbacks underscore the need for alternative treatments that can overcome these challenges and provide more effective and safer options for cancer patients. In recent years, the use of live bacteria, engineered bacteria, or bacterial derivatives to deliver antitumor drugs to specific tumor sites for controlled release has emerged as a promising therapeutic tool. This approach offers several advantages over traditional cancer therapies, including targeted drug delivery and reduced toxicity to healthy tissues. Ongoing research in this field holds great potential for further developing more efficient and personalized cancer therapies, such as E. coli, Salmonella, Listeria, and bacterial derivatives like outer membrane vesicles (OMVs), which can serve as vehicles for drugs, therapeutic proteins, or antigens. In this review, we describe the advances, challenges, and future directions of research on using live bacteria or OMVs as carriers or components derived from bacteria of delivery systems for cancer therapy.

## 1 Introduction

Based on the “Global Cancer Statistics 2020” released by the American Cancer Society, cancer is a significant human health threat in developed and developing countries. It is increasingly becoming a leading cause of death, with over half of all cases occurring in Asia (Sung et al., 2021). Existing cancer treatments include chemotherapy, radiation therapy, surgery, and biologic therapies (Lou et al., 2021). Chemotherapy is a systemic treatment that can kill tumor cells but may also harm normal cells, leading to side effects and a high risk of drug resistance (Liang et al., 2019). Radiotherapy is a localized treatment, but it may not reach certain parts of the tumor, and some tumors are not sensitive to radiation therapy, resulting in treatment failure. Immunotherapy, which includes immune checkpoint inhibitors, tumor vaccines, CAR-T cell therapy, and others (Waldman et al., 2020), typically has fewer toxic side effects than traditional antitumor therapies. However, the therapeutic effect of single immunotherapy is often limited, and it is usually combined with radiotherapy and chemotherapy to enhance tumor treatment effectiveness (Cao and Liu, 2020). Therefore, there is an urgent need to investigate new therapeutic approaches for tumor control.

**FIGURE 1:**
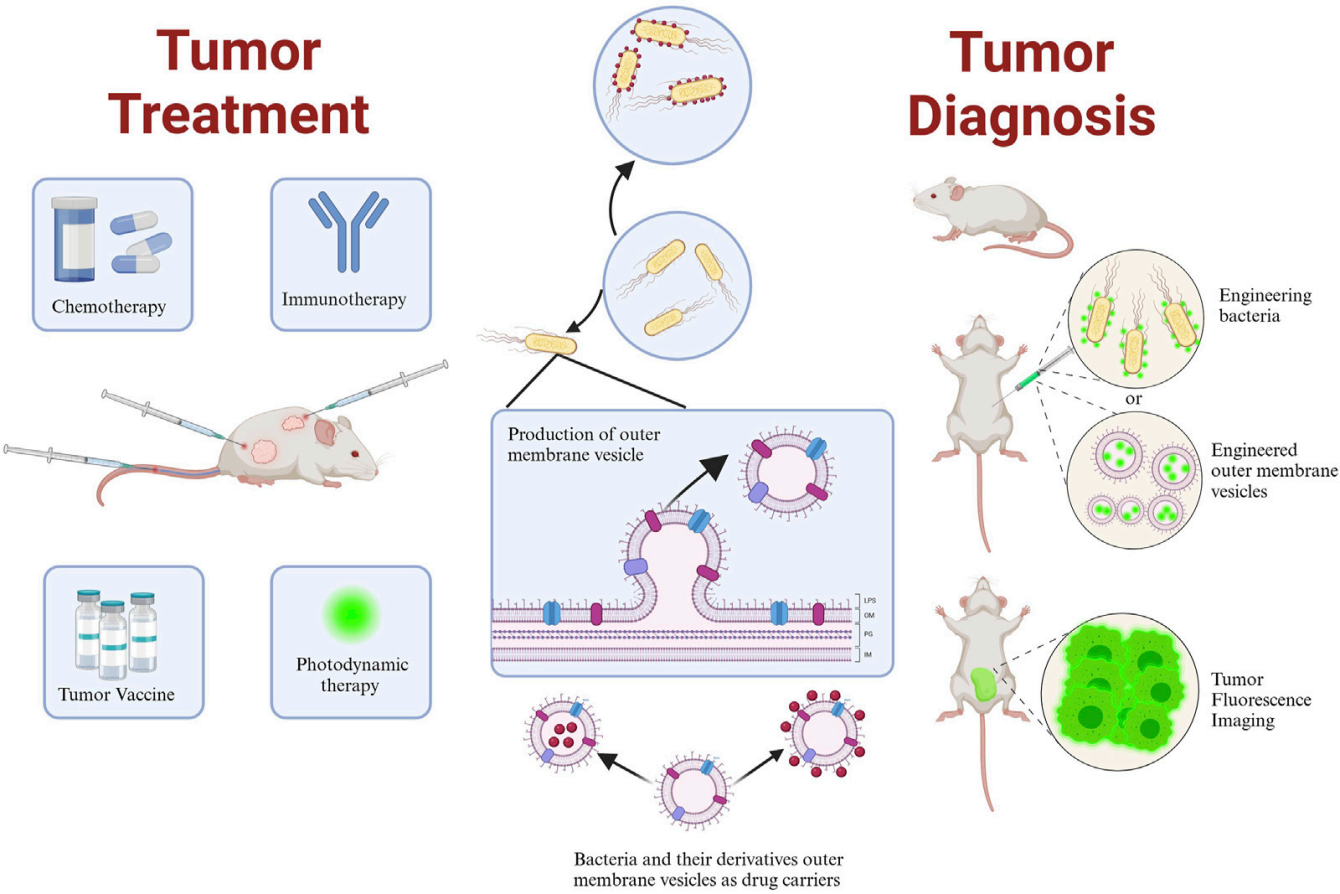
Schematic representation of the application of bacteria and their derivative outer membrane vesicles in tumor therapy and diagnosis. Created with BioRender.com.

Bacteria are organisms that have existed for millions of years and are essential for human health due to their unique functions, such as motility, microenvironment sensing, nutrient competition, intra-tumor targeting, the presence of extracellular enzymes, and immune stimulation (Dróżdż et al., 2020). Additionally, some anaerobic bacteria can survive and multiply under hypoxic or micro-hypoxic conditions. Facultative anaerobes, including Salmonella, are highly adaptable, can colonize aerobic and anoxic environments, produce ATP aerobically or anaerobically and promote the migration (Dróżdż et al., 2020). This ability enables the effective control of antitumor loads at the tumor site and precise timing of drug transport while reducing systemic toxicity to the host by regulating the expression of bacterial genes (Yang et al., 2021). Based on this, certain facultative anaerobes, such as *Escherichia coli* (Jiang et al., 2010), *Salmonella* (Liang et al., 2019), and *Listeria monocytogene* (Yang et al., 2014)*,* have been exploited or genetically modified to become tumor-targeting bacteria for delivering cytokines, immunomodulators (Chowdhury et al., 2019), cytotoxic agents (Li et al., 2019), therapeutic peptides/proteins (Chiang and Huang, 2021), prodrug-converting enzymes (Aganja et al., 2022), and small interfering RNA to the tumor site (Yang et al., 2021), moreover, bacteria’s capacity to produce outer membrane vesicles (OMVs) has been utilized for tumor immunity, tumor-engineered vaccines, and drug delivery, making it a novel tool with great potential for antitumor therapy and biological applications (Figure 1).

In recent years, with the advancements in molecular biology and nanotechnology, there have been significant developments in the design of advantageous components derived from bganxieninde1acteria. These advancements include the development of bacterial membrane-encapsulated nano-drug delivery systems and bacterial-nanoparticle hybridization systems. These innovations not only promote the application of bacteria but also offer new opportunities for optimizing protocols in tumor treatment (Cacicedo et al., 2018; Hou et al., 2021; Li et al., 2022; Wang et al., 2022; Xie et al., 2022). This article aimed to summarize the genetic editing or engineering techniques used in several facultative anaerobes and other bacteria for antitumor therapy and to discuss the potential future directions in drug delivery vehicles.

## 2 Facultative anaerobes-based drug delivery systems

**FIGURE 2:**
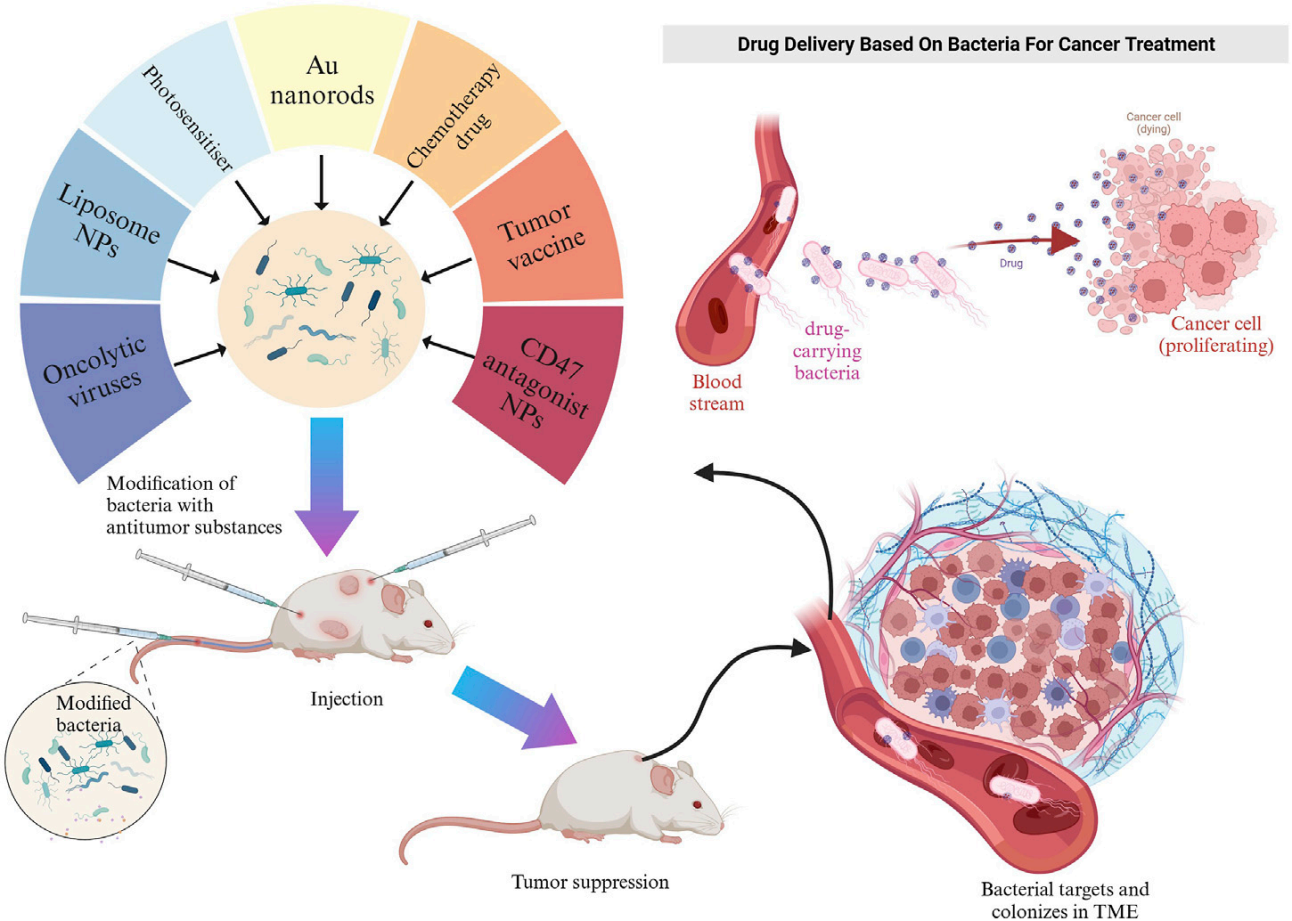
Schematic representation of engineered bacteria target TME in mice. Created with BioRender.com.

Genetically engineered bacteria can be utilized as vectors for tumor treatment or immune system activation (Figure 2) (Fu et al., 2023). Their ability to colonize tumors as drug-delivery vehicles depends on the conditions and composition of the tumor microenvironment (TME). The TME is a complex multicellular environment that includes soluble factors, transformed extracellular matrix, epigenetically modified and reprogrammed fibroblasts, as well as immunosuppressive cells (Deepak et al., 2020). The TME is characterized by hypoxia and low pH values, primarily caused by abnormal capillary formation leading to hypoxia and nutrient deficiencies in specific areas. Cellular metabolism also produces significant amounts of lactic acid (Zhang et al., 2022). Both the high-lactate and low-nutrient properties of the TME are compatible with the growth conditions of facultative anaerobes. Additionally, hypoxia provides the most common mechanism for bacterial targeting of tumors (Holay et al., 2021). Consequently, the colonization of anaerobic bacteria can be limited to the hypoxic TME without affecting healthy tissues (Zhou et al., 2018; Holay et al., 2021). As drug delivery agents, facultative anaerobes can increase the local drug concentration in the TME, reduce drug extravasation to surrounding healthy tissues, and mitigate drug side effects. This section introduced some classical facultative anaerobes and other bacteria as drug carriers (Table 1).

**TABLE 1:** Bacteria as a vehicle for target delivery in cancer therapy.

*Type of bacteria*	*Type of engineering modifications*	*Delivered substance*	*Efficacy*	*References*
***NiCo21(DE3) E. coli (NEB)***	synchronous cracking circuit	CD47 antagonist NPs	Increased activation of T cells	Chowdhury et al. (2019)
***E.coil ATCC 25922***		liposomal paclitaxel (LP)	Inhibited the proliferation of lung cancer cells	Zhang et al. (2020)
***E.coil(NEB)***	Expression of human peroxidase (HPO)	pDA/Ce6 NPs	Enhanced PTT	Deng et al. (2021)
***E.coil BL21***		bovine serum albumin	Enhanced adhesion and targeting of cancer cells	He et al. (2023a)
***E.coil BL21***		liposomes encapsulated with oncolytic viruses	Increased enrichment and infiltration of oncolytic viruses and immune cells in the tumor region	Sun et al. (2022)
***E.coil BL21***		p53 protein and anti-angiogenic factor Tum-5	Anti-tumor effect	He et al. (2019)
***E.coil BL21***	Overexpression of glucose dehydrogenase	DOX	Reduced toxicity of DOX and enhanced efficacy of DOX-mediated immunotherapy and chemotherapy	Xie et al. (2017)
***E.coil BL21***	Expression of HSulf-1	Adriamycin NPs	Tumor cell DNA damaged and inhibits melanoma and reduces side effects	Yang et al. (2023)
***E.coil BL21***	Produce GVs, and carry ARGs	Multifunctional Cationic Lipids NPs	Enhances the efficacy of FUAS and stimulates AQ4N and FUA to work together to kill tumor cells	Wang et al. (2022d)
***E.coil BL21***	Produce GVs, and carry ARGs		Inhibits the growth of breast tumors	Yang et al. (2021a)
***Nissle 1917***		TeNRs	Induces tumor cell damage and death, remodels TME, and inhibits tumor recurrence and metastasis	Yao et al. (2022)
***Nissle 1917***	Produces butyrate		Reduced side effects and increased bioavailability	Chiang and Hong (2021)
***Nissle 1917***	ara BAD promoter	Cytotoxic small protein Hly E	Tumor cells from colorectal cancer in mice subsided	Chiang and Huang (2021)
***Nissle 1917***		DOX	Achieved acid-responsive release of anticancer drugs improves the antitumor efficacy of intravenously administered Ec N-ca-DOX	Xie et al. (2017)
***Nissle 1917***		5-FU, ZOLs and Au nanorods	Achieved controlled release of drugs to inhibit tumor growth	Xie et al. (2021)
***Nissle 1917***		5-ALA	Antitumor protoporphyrin X (PpⅨ) accumulates in tumors without harming normal tissue cells	Chen et al. (2021a)
***Nissle 1917***	Expressed peroxidase	BPQDs	Enhanced PDT	Ding et al. (2021)
***MG1655***	Expressed TNF	BPNPs	Promote tumor cell apoptosis, activate T-lymphocytes, promote the release of inflammatory factors	Chen et al. (2023a)
***MG1655***	Expressed Cly A	AuNPs	Enhanced PTT and inhibited tumor cell proliferation	Wang et al. (2021)
***MG1655***		R848 and DOX NPs	Enhanced IL-6 secretion and anti-tumor immune response, weakened TME immunosuppression, remodeled TME, and triggered immunogenic cell death	Wei et al. (2021)
***MG1655***	Expressed tyrosinase		Improved visualization of cancer-targeting bacteria	Yun et al. (2021)
***Salmonella typhimurium***	Expressed anti-TNF-α antibody	mCherry	Inhibited melanoma tumor progression and promoted anti-tumor immune responses in the melanoma TME	Liu et al. (2023a)
***Salmonella typhimurium***	Expressed Cly A		Destroyed tumor stromal cells and cancer cells, promoted the secretion of interleukin (IL-1b), TNF - α cytokines, and inhibited tumor growth	Tan et al. (2022)
***Salmonella typhimurium***		ICG NPs	Tumor cells were killed using the photothermal effect of INPs	Chen et al. (2019)
***Salmonella typhimurium***		Co-expression plasmid (pcDNA3.1-HPV16-L1-siE6)	Produced HPV16-L1 antibodies INF-γ and IL-2, which silenced the E7 and E6 genes and inhibited the growth of cervical cancer	Chen et al. (2021b)
		Neuroblastoma DNA Vaccine	Stronger immune response	Stegantseva et al. (2020)
***Salmonella typhimurium***	Expressed Bax BH3 polypeptide		Reduced survival and apoptosis of human B-type NHL cell line Ramos cells	Mateos-Chávez et al. (2019)
***Listeria monocytogenes***		Immunogenic tetanus toxoid protein (TT856-1313)	Production of anti-tumor substances reduced pancreatic tumor load and metastatic foci and improved survival in mice	Selvanesan et al. (2022)
***Listeria monocytogenes***	Expressed ANXA2		Increased expression of interferon-gamma (INFγ) in TME prolongs survival of mice with pancreatic ductal adenocarcinoma	Kim et al. (2019)
***Listeria monocytogenes***	actA and plcB gene deletion, shuffled HPV-16 E6E7 protein recombination		Prevented cervical cancer	Su et al. (2021)
***Bifidobacterium bifidum***		ICGNPs	Enhancement of PTT	Reghu and Miyako (2022)
***Bifidobacterium bifidum***		Ce6 NPs and anti-death receptor 5 antibody	Inhibited tumor growth	Li et al. (2022b)
***Bifidobacterium bifidum***		DOX and CaP/SiO2 NPs (DNPs)	Induced ICD and inhibited primary tumor while suppressing melanoma metastasis in mice	He et al. (2023b)
***Akkermansia muciniphila***			Helped suppress colorectal tumors and predict clinical responses to immune checkpoint inhibitors in non-small cell lung cancer and advanced melanoma	Derosa et al. (2022), Lee et al. (2022)

### 2.1 *E. coli* as drug delivery vehicles

**FIGURE 3:**
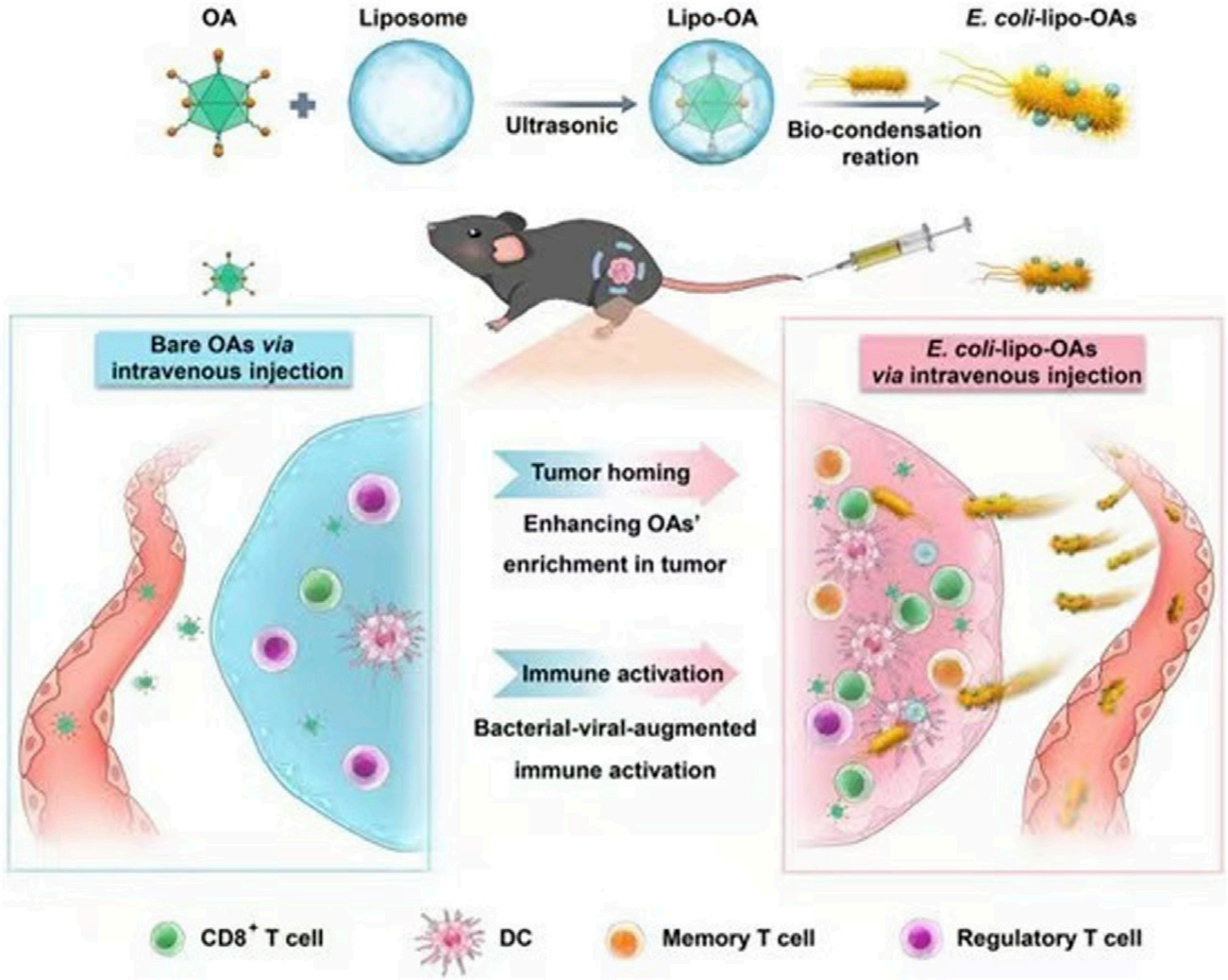
Schematic representation of self-propelled bacterium-vessels carrying OAs that can home to the tumor lesion and enhance the antitumor responses through bacterial–viral-augmented immune activation. Reproduced with permission from (Sun et al., 2022). Copyright ^©^ 2022 American Chemical Society.

*E. coli* is a facultative anaerobe that can colonize and proliferate in hypoxic or necrotic regions of tumors (Xie et al., 2022). Based on this property, genetically modifying them to carry drugs, cytokines, etc., can lead to antitumor ability, targeting, or production of beneficial substances for tumor therapy (Zhang et al., 2021; Howell and Forbes, 2022). He et al. modified Bovine serum albumin on the surface of attenuated *E. coli*. They demonstrated that modified E. coli enhanced adherence to and targeted cancer cells, with more vital adherence and targeting of bladder cancer cells with high expression of secreted acidic cysteine-rich proteins (He et al., 2023). Oncolytic viruses can regulate the TME and optimize the effects of immune-mediated tumor eradication (Harrington et al., 2019). However, as a monotherapy, their efficacy is more limited (Fukuhara et al., 2016). Sun et al. encapsulated a layer of biocompatible and biopreservation liposomes on the surface of the oncolytic viruses and then constructed “*E. coli* liposome-OA.” Thanks to the high homing ability of *E. coli*, the enrichment rate of lysosomal viruses in the tumor was significantly increased, and the inflammatory response of *E. coli* in the tumor region was utilized to enhance the infiltration of immune cells in the tumor region (Figure 3) (Sun et al., 2022). Zhang et al. used electroshock to transfer prepared paclitaxel liposomes into *E. coli* or *Lactobacillus casei*, and found that the *E. coli*-containing preparation had a more potent anticancer effect than the *L. casei*-containing preparation (Zhang et al., 2020). CD47 is a transmembrane protein expressed in phagocytes and abundantly expressed in various malignant cells. CD47 blocks the drive for T cell-mediated immune tumor eradication. The antitumor activity of anti-CD47 therapies is mediated by blocking antiphagocytic signaling (Liu et al., 2015). Sreyan et al. designed *E. coli* strains containing synchronous lysis circuits to colonize tumors with CD47-releasing nano-antibody antagonists, increasing the activation of tumor-infiltrating T cells and inducing persistent and systemic anti-tumor immunity (Chowdhury et al., 2019). Notably, the ability of *E. coli* itself to act as a foreign substance to stimulate the immune system and enhance anti-tumor immunity is also a significant advantage as a drug delivery vehicle (Sun et al., 2022).

As a highly proliferative tissue, tumor tissue requires neovascularization to transport oxygen, nutrients and excrete metabolic waste (Eulberg et al., 2022). Therefore, tumor progression necessitates the initiation of tumor angiogenesis (Lugano et al., 2020). Conversely, inhibiting tumor angiogenesis or destroying existing vessels can slow tumor progression. Heparan sulfatase 1 (HSulf-1) suppresses tumorigenesis by inhibiting angiogenesis *in vivo* (Narita et al., 2006). Yang et al. demonstrated that the modification of *E. coli* to express HSulf-1 (HSEc) *in vivo* resulted in the overexpression of HSulf-1. HSEc can actively target and colonize tumor sites to hinder angiogenesis and metastasis. Moreover, the surface-attached adriamycin glycogen nanoparticles (NPs) can penetrate tumor cells, leading to intracellular DNA damage (PDOX@). The combined biological and chemotherapeutic treatment with HSEc shows significant inhibition of melanoma and reduced side effects in mouse models (Yang et al., 2023).

*E. coli*, when genetically modified, can be combined with other therapeutic tools to achieve antitumor effects (Lou et al., 2021). For instance, Sun, Ye et al. modified *E. coli* to overexpress glucose dehydrogenase. Subsequently, doxorubicin (DOX) was coupled to the surface of the modified *E. coli*, reducing the toxic effect of DOX through the self-driven tumor colonization property of *E. coli*, resulting in its selective accumulation in tumors. Meanwhile, DOX activated NADPH oxidase and catalyzed the conversion of NADPH and O_2_ to NADP+ and reactive oxygen species (ROS), enhancing the efficacy of DOX-mediated immunotherapy and chemotherapy. In a mice tumor model, the modified bacteria inhibited the growth of primary and distant tumors and synergistically enhanced the efficacy of immunotherapy (Ushio-Fukai and Nakamura, 2008; Kan et al., 2014; Skonieczna et al., 2017; Sun et al., 2021). Deng et al. genetically modified non-invasive *Escherichia coli* to obtain *E. coli* overexpressing human peroxidase coli(p). Under near-infrared (NIR) light irradiation, the generated O_2_ was converted to cytotoxic ^1^O₂. PDA/Ce6 was obtained by encapsulating polydopamine (pDA) with chlorin e6 (ce6), and then pDA/Ce6 was encapsulated with *E. coli*(p) to obtain *E. coli*(p)/pDA/Ce6. Coli(p)/pDA/Ce6 was replicated and accumulated in tumors, and NIR light was introduced to achieve photothermal and O_2_-enhanced photodynamic therapy (PDT) (Deng et al., 2021). In another study conducted by Dai et al., various charges of zinc phthalocyanine (ZnPc) photosensitizers were prepared and investigated for their electrostatic adherence characteristics to *E. coli*. The researchers found that ZnPc conjugates with four positive charges (designated as ZnPc-IR710) demonstrated the most extraordinary capacity for loading and the best imaging properties of fluorescence to *E. coli*. With the support of *E. coli*, *E. coli* @ ZnPc - IR710 significantly inhibited tumor growth in a mouse subcutaneous 4T1 breast cancer model (Dai et al., 2020). Wang et al. genetically engineered *E. coli* to produce gas vesicles, known as GVs-*E. coli*, which carried an acoustic reporter gene (ARG) encoding the formation of gas vesicles (GVs). They performed ultrasound imaging and co-focused ultrasound ablation surgery (FUAS) for hypoxic tumor regions and environments in mice. The researchers enriched multifunctional NPs in tumor target areas and co-delivered multifunctional cationic lipid NPs (including IR780, perfluorohexane, and banoxantrone dihydrochloride (AQ4N)) to tumors, enabling focused multimodal imaging and enhancing the efficacy of FUAS. The hypoxic environment of the tumors stimulated AQ4N to kill tumor cells in conjunction with the FUAS procedure (Wang et al., 2022). Similarly, experiments by Yang and Jiang et al. demonstrated that genetically modified *E. coli*, carrying an ARG encoding the formation of GVs, could be visualized using ultrasound. Intravenous injection of the modified *E. coli* for targeted breast cancer therapy specifically targeted tumor areas and persistently localized into the TME, resulting in significant suppression of tumor growth (Yang et al., 2021).

#### 2.1.1 *E. coli* nissle 1917

The well-studied *E. coli* Nissle 1917 (Ec N) is a non-pathogenic probiotic that selectively colonizes the intermediate zone between solid tumors’ live and necrotic zones (Li et al., 2019). It is difficult for chemotherapeutic agents to cross the biological barrier to enter solid tumors’ necrotic and hypoxic zones, whereas Ec N can selectively proliferate in these zones. This property makes it a high-quality vehicle for drug delivery (Chen et al., 2023). At the same time, it was found that Ec N also has a significant advantage over conventional nanomedicines in tumor accumulation and overall biodistribution, and the average amount of Ec N detected in tumors is remarkably higher than the number of injections due to the fixation and multiplication of Ec N (Liu et al., 2021). However, using NPs and other decorative drug surfaces can increase the stability of the drug *in vivo* and prolong the residence time of the drug in the circulatory system. Therefore, nanomaterials can be combined with Ec N to overcome the problem of small cumulative doses of nanomedicine in the tumor region and achieve tumor-targeted therapy. Yao et al. utilized Ec N and synthesized TeNRs intracellularly to obtain Te@EcN, an integrated therapeutic system with a high loading rate and efficient photothermal conversion, which can damage and induce tumor cell death after irradiation with NIR. Meanwhile, Ec N could act as an immune adjuvant to promote dendritic cells (DCs) maturation and further enhance the initiation of cytotoxic T cells. Te@EcN reprogrammed tumor-associated macrophages (TAMs) and remodeled the immune-suppressive TME, which inhibited tumor recurrence and metastasis (Yao et al., 2022).

Ec N can also be modified to produce or carry desired anticancer substances, including butyrate, a short-chain fatty acid produced by colon intestinal bacteria fermenting dietary fiber, known for its ability to inhibit the proliferation of cancer cells (Hajjar et al., 2021; Richter et al., 2021). Deng et al. genetically modified Ec N to synthesize bio butyrate under hypoxic conditions, eliminating the need for butyrate purification and delivering butyrate *in situ*, thereby mitigating side effects and improving bioavailability. Moreover, the bio butyrate-producing E. coli can also deliver other anticancer drugs or therapeutic adjuvants, further enhancing the therapeutic efficacy of the delivery system *in vivo* (Chiang and Hong, 2021). It was reported that under the control of the ara BAD promoter (PBAD), which enables Ec N to contain the small cytotoxic protein Hly E, the expression of HlyE was efficiently regulated in a time-controlled manner, resulting in specific colonization of tumors with a tumor-organ ratio of 106:1 and causing tumor cell regression in murine colorectal cancer (Chiang and Huang, 2021). In another study, He et al. utilized Ec N as a carrier to transport the anticancer protein p53 and the antiangiogenic factor Tum-5 to the tumor hypoxic zone in cancer treatment, which yielded excellent tumor treatment results (He et al., 2019). Additionally, Xie et al. attached DOX to Ec N using an acid-soluble cis-aconitic anhydride linker (Ec N-ca-Dox) to achieve targeted accumulation of bacteria in tumors and acid-responsive release of anticancer drugs. In an experiment where Ec N-ca-Dox was intravenously injected into mice for 3 hours, the accumulation of DOX in tumors was 12.9% per Gram of tissue injected dose, which decreased to 6.4% after 3 days. This approach proved to be far superior to nanocarriers, significantly improving the antitumor efficacy of intravenous Ec N-ca-Dox treatment by inhibiting tumor growth, prolonging animal survival, and inducing apoptosis of tumor cells (Xie et al., 2017).

Tumor treatments using Ec N as a drug carrier can also be combined with other treatments, such as chemotherapy, immunotherapy, and photothermal effects, to improve therapeutic outcomes and minimize side effects (Li et al., 2019; Wang et al., 2022; Yao et al., 2022; Zhu et al., 2023). For instance, Xie et al. specifically developed Ec NZ/F@Au by incorporating fluorouracil (5-FU) and macrophage phenotypic modulators zoledronic acids (ZOLs) into Ec N through electroporation and modifying gold nanorods on its surface. When exposed to NIR irradiation, the photothermal effects of the gold nanorods increased the local temperature and facilitated the *in-situ* conversion of active Ec N into bacterial ghosts (BGs). This conversion led to a gradual release of the drug from BGs through transmembrane channels, while intermittent NIR light triggered a stepwise increase in BGs formation and drug release, enabling controlled drug release. The combined action of 5-FU and ZOLs effectively inhibited tumor growth (Xie et al., 2021). Photosensitizers are crucial in PDT. One example is 5-Aminolevulinic acid (5-ALA), an intelligent photosensitizer that can be converted into protoporphyrin IX (Pp IX), a potent photosensitizer for PDT (Kim et al., 2013; Chen et al., 2021a). Based on this, Chen et al. employed Ec N as a carrier for 5-ALA delivery to colorectal cancer (CRC) cells, which induced the aggregation of anti-tumor protoporphyrin X (PpⅨ) under 630 nm laser irradiation. The results demonstrated the effectiveness of this administration method without causing harm to normal tissue cells (Chen et al., 2021a). O_2_ is a crucial component of PDT (Agostinis et al., 2011). Ding et al. genetically modified Ec N to express peroxidase and created an engineered *E. coli*/BPQDs (EB) system by attaching black phosphorus quantum dots (BPQDs) to the bacterial layer through electrostatic attraction. This system was capable of targeting hypoxic tissues after intravenous injection and, upon irradiation with a 660 nm laser, generating reactive oxygen species (ROS) and disrupting cell membranes. Consequently, peroxidase was released, degrading hydrogen peroxide to produce O_2_ and enhancing PDT (Ding et al., 2021). It is not difficult to find that when antitumor therapy using Ec N as a drug carrier is combined with other antitumor therapies, it can produce synergistic and surprising therapeutic effects.

#### 2.1.2 *E. coli* MG1655

The nonpathogenic MG1655 naturally reduces nontoxic nitrate to nitrous oxide with antitumor activity through nitrate/nitrite reductase (Gusarov et al., 2009; Zheng et al., 2018). It is frequently utilized in antitumor therapy as an engineered bacterium, combined with other therapeutic approaches such as immunotherapy and photothermal therapy (PTT). Chen et al. engineered MG1655 to express tumor necrosis factor and bind it to black phosphorus NPs. This binding facilitates the transfer of photoelectrons generated by laser irradiation of black phosphorus NPs, triggering the metabolism of nitrate reduction to nitric oxide for *in-situ* release at the tumor site. This process promotes therapeutic efficacy and the polarization of M2-type macrophages to M1-type macrophages. Simultaneously, the immune system enhances the immune effect by i) promoting apoptosis of tumor cells, ii) activating the action of T lymphocytes, and iii) promoting the release of pro-inflammatory factors (Chen et al., 2023). Wang et al. biomineralized gold NPs in MG1655 and constructed a thermally activated biohybrid system (TAB@Au) by exploiting the bacteria’s ability to express the therapeutic protein cytolysin A (Cly A) (Wai et al., 2003; Wang et al., 2021). After laser irradiation of the gold NPs, the collected photons are converted into heat, and the inserted promoter is used to induce the expression of Cly A, thereby enhancing the PTT and inhibiting the proliferation of tumor cells (Wai et al., 2003; Wang et al., 2021). Wei et al. prepared Riquimod (R848) and DOX NPs, which attached *E. coli* to R848 through electrostatic action to form R848-loaded *E. coli*. This system targets the hypoxic environment of tumors, and the *E. coli* is phagocytosed by M2-type macrophages and releases R848, polarizing the M2-type macrophages to M1-type macrophages. This process enhances the secretion of cytokines such as IL-6, promotes anti-tumor immune response, remodels the immunosuppressed TME, and kills tumor cells. DOX NPs can trigger immunogenic cell death, weakening the immunosuppression of the TME and promoting immunotherapy (Wei et al., 2021).

*E. coli* MG1655, an engineered bacterium, can be utilized for precise diagnosis and prediction of treatment response. According to Yun et al., optoacoustic images and tyrosinase-expressing *E. coli* were employed to enhance the visualization of cancer-targeting bacteria, enabling accurate diagnosis and prediction of treatment response in colon cancer cases (Yun et al., 2021).

### 2.2 *Salmonella* as drug delivery vehicles

*Salmonella typhimurium*, a facultative anaerobe, is capable of surviving in both aerobic and anaerobic environments. It has the ability to target and colonize both non-hypoxic and hypoxic tumors (Nguyen and Min, 2017). It replicates preferentially in tumor areas, with a ratio of up to 1000:1 or even 10,000:1 compared to normal tissue (Eisenstark, 2018). Based on these properties, *S. typhimurium* has been designed and engineered as a targeted treatment for cancer. It can be combined with other therapies, such as chemotherapy and radiotherapy, to enhance their effects, synergistically modulating the TME and offering significant diagnostic and therapeutic advantages (Mi et al., 2019). *S. typhimurium* can be modified to transport antitumor substances and induce tumor regression (Zhao et al., 2005; Zhao et al., 2006). This section focused on the experimental progress of *Salmonella typhimurium* as a vector for tumor treatment and diagnosis.

**FIGURE 4:**
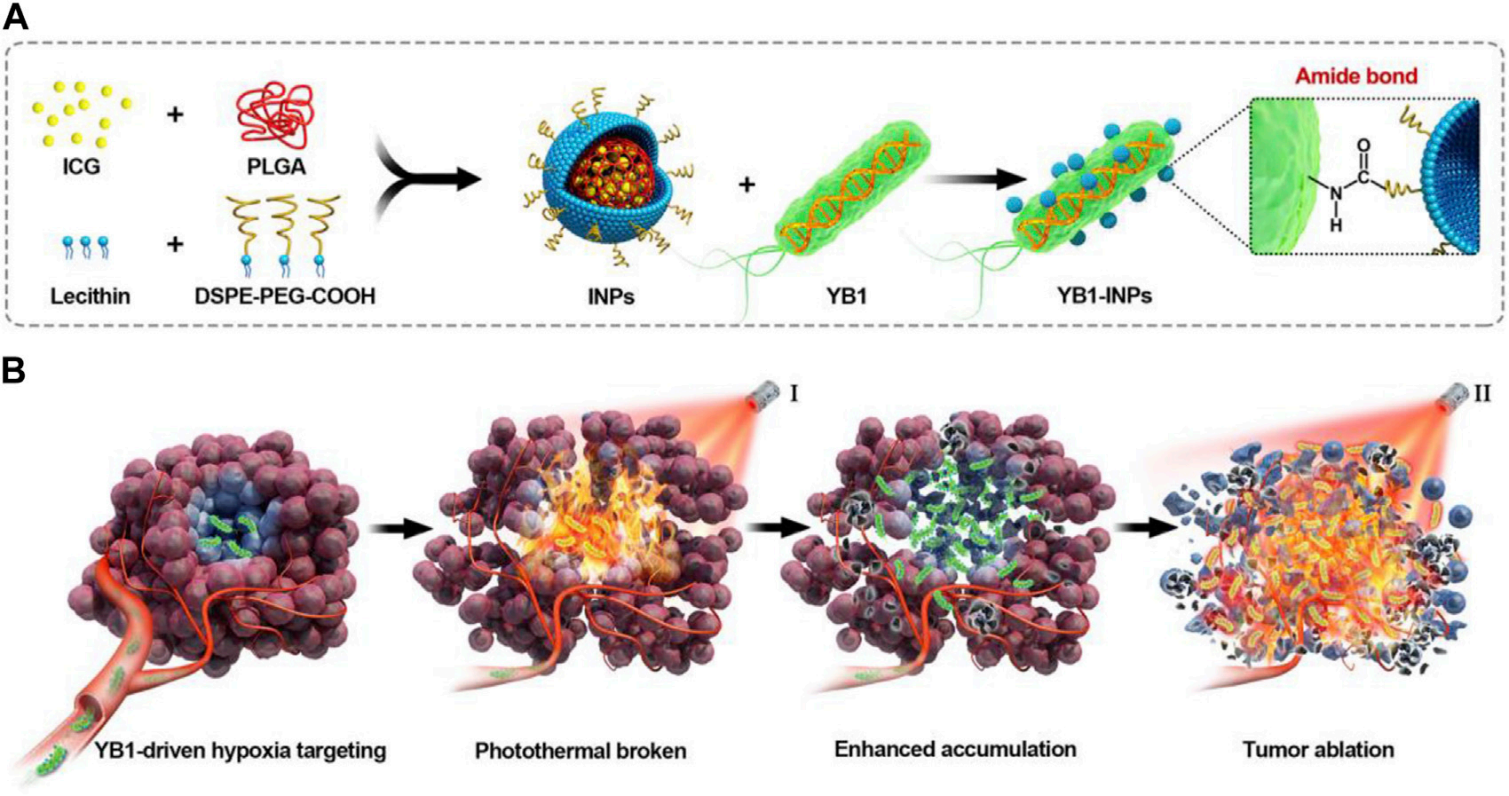
Schematic illustration of nanophotosensitizers (INPs)-attached YB1 as a hypoxia-targeting delivery system for large solid tumors photothermal therapy. **(A)** Preparation procedure of YB1-INPs. Synthesized INPs with single-step sonication were attached to YB1 through amide bonds. **(B)** YB1-INPs with hypoxia-targeting and photothermal-assisted bioaccumulation for tumor penetrative therapy. After migrating into tumor hypoxic cores and subsequently irradiating with NIR laser, the loosening of tumor tissue and tumor lysis generate bacteria-attracting nutrients, which further enhances the accumulation and coverage of YB1-INPs in large solid tumors. Ultimately, the enriched YB1-INPs under NIR laser irradiation completely ablated the large solid tumor without relapse. Reproduced with permission from (Chen et al., 2019). ^©^ 2019 Elsevier Ltd. All rights reserved.

The VNP20009 gene modification strain (VNP) can be enriched in tumors due to its extremely tumor-targeting properties and stable heritability (Liu et al., 2023). Tumor necrosis factor α (TNF-α) is a tumor necrosis factor that can cause tumor necrosis with high doses and repeated local injections (Balkwill, 2009). However, the significant toxicity to humans limits the use of TNF-α. Liu et al. constructed a new VNP delivery system that expresses anti-TNF-α nanoantibodies, which can dramatically increase delivery efficiency by continuously releasing nanoantibodies in a hypoxic tumor environment. They combined the delivery system with cherry to visualize tumor-expressed TNF-α nb. It was demonstrated that the system showed sound therapeutic effects in melanoma mice, which may be related to the fact that VNP α TNF-α inhibited tumor progression by reducing tumor vascular density, inducing more apoptosis in tumor tissues, and activating the immune system (Liu et al., 2023). Tan et al. engineered *S. typhimurium* to express Cly A by genetic modification for treating mouse pancreatic cancer. It was demonstrated that after intravenous injection, the engineered bacteria could colonize the TME, significantly reduce the expression of tumor mesenchymal cell markers, increase immune cells, and destroy tumor stromal cells and cancer cells, thereby significantly promoting the secretion of inflammatory cytokines, such as interleukin (IL-1b) and TNF-α, and inhibiting tumor growth (Tan et al., 2022). Chen et al. developed indocyanine green (ICG)-loaded NPs (INPs) as nano photosensitizers for fluorescence-guided PTT of tumors, followed by attaching INPs to *S. typhimurium* YB1 and delivering them to the oxygen-deficient tumor cores to kill tumor cells with the photothermal effect of the INPs (Figure 4) (Chen et al., 2019). INPs are highly biocompatible, have high spatiotemporal selectivity, and excellent photothermal conversion efficiency (Zheng et al., 2013; Chen et al., 2016; Chen et al., 2019). Under NIR laser irradiation, the temperature of the tumor region can reach up to 63 °C, killing both the tumor cells and the YB1 strain colonized within the tumor (Chen et al., 2019).

Attenuated *Salmonella* can also be utilized for delivering antitumor antigens. In the experiments conducted by Chen et al., they constructed a plasmid (pcDNA3.1-HPV16-L1) and a co-expression plasmid carried by *Salmonella* attenuata (pcDNA3.1-HPV16-L1-siE6). The *Salmonella* carrying pcDNA3.1-HPV16-L1 can generate HPV16-L1 antibodies INF-γ and IL-2 in the serum of mice, which have a preventive effect against HPV. Furthermore, pCG-siE6 suppressed the expression of E7 and E6 genes, leading to the inhibition of cervical cancer growth both *in vitro* and vivo (Chen et al., 2021b). Maria V. Stegantseva et al. examined the delivery method of DNA vaccines for neuroblastoma and discovered that trans-*Salmonella* delivery of DNA vaccines elicited a stronger immune response (Stegantseva et al., 2020).

Resistance to chemotherapeutic agents often leads to poor tumor treatment or tumor recurrence (Zahan et al., 2020). One resistance mechanism that is strongly linked to modifying genes and proteins regulating the apoptotic mitochondrial pathway is members in the Bcl-2 family (Stavrovskaya, 2000; Johnstone et al., 2002; Mateos-Chávez et al., 2019). The BH3 structural domain peptide from the proapoptotic protein Bax has the potential to oppose the anti-apoptotic activities of proteins in the Bcl-2 family, thereby recovering apoptosis and inducing chemo-sensitivity in tumor cells (Moreau et al., 2003; Mateos-Chávez et al., 2019). In a study by Alfredo Mateos-Chávez et al., the Bax BH3 polypeptide was expressed and released using the Mis L self-transport system on the surface of *S. typhimurium* SL326183 (Mateos-Chávez et al., 2019). The results showed that *Salmonella* L-STXP significantly reduced the viability and induced apoptosis in the human B-type non-Hodgkin’s lymphomas (NHL) cell line Ramos cells. These findings have positive implications for the remission of non-Hodgkin’s lymphoma and the reduction of drug resistance (Mateos-Chávez et al., 2019).

### 2.3 *Listeria monocytogenes* as drug delivery vehicles

Unlike *E. coli* and *Salmonella*, *Listeria monocytogenes* (LM) is a Gram-positive bacterium with a unique life cycle (Oladejo et al., 2021). A portion of these LM escapes from primary phagocytic vesicles, grows and divides macrophages in the cytoplasm, provides cell-penetration drive via host cell actin and filamentous pseudopod protrusions, and repeats the cycle of growth, actin recruitment, and cell-to-cell spread (Goldfine and Wadsworth, 2002). Infected LM and its progeny can bypass an organism’s humoral immune system (Brunt et al., 1990). The cell-mediated immunity of LM is associated with CD4^+^ and CD8^+^ T-cell responses, and it is this feature that makes LM ideally suited to serve as a platform for antigen delivery (Goldfine and Wadsworth, 2002; Oladejo et al., 2021). This section presented recent experimental advances in LM as an antitumor delivery platform.

Pancreatic tumors are located in a relatively hidden manner, and early symptoms are not easily detectable. However, these tumors have a high metastasis rate and often result in a poor prognosis. Therefore, there is an urgent need to develop methods for effective preventive control. In a study by Benso et al., immunogenic tetanus toxoid proteins (TT856-1313) were selectively delivered into pancreatic ductal tumor cells using attenuated LM. This led to the accumulation of tetanus toxoid proteins within the tumor cells, introduction of tetanus toxoid protein CD4 T cells into the TME, and production of anti-tumor substances. As a result, tumor load and metastatic foci were effectively reduced, and survival rates in mice increased (Selvanesan et al., 2022). Another study by Victoria M. Kim et al. focused on LM immunotherapy based on ANXA2 expression (Lm-ANXA2). It was discovered that Lm-ANXA2 significantly prolonged survival in a mouse model of PDACs. Furthermore, sequential treatment with Lm-ANXA2 and anti-PD-1 increased interferon-γ (INFγ) expression in the TME and promoted ANXA2 epitope formation. These findings suggest that LM has the potential to serve as a vector in the treatment of pancreatic tumors (Kim et al., 2019). In addition to pancreatic tumors, some researchers have identified a potential role for immunotherapy in the treatment of cervical cancer. Su et al. genetically modified LM by deleting the actA and plcB genes or recombining shuffled HPV-16 E6E7 protein expression. They then delivered these modified strains to the spleen and liver through intravenous injection, inducing antigen-specific cellular immunity. The study experimentally demonstrated the effectiveness of both genetically modified strains against cervical cancer. It was found that combined immunotherapy was more effective than single-strain immunotherapy (Su et al., 2021).

### 2.4 *Bifidobacterium* bifidum as drug delivery vehicles

**FIGURE 5:**
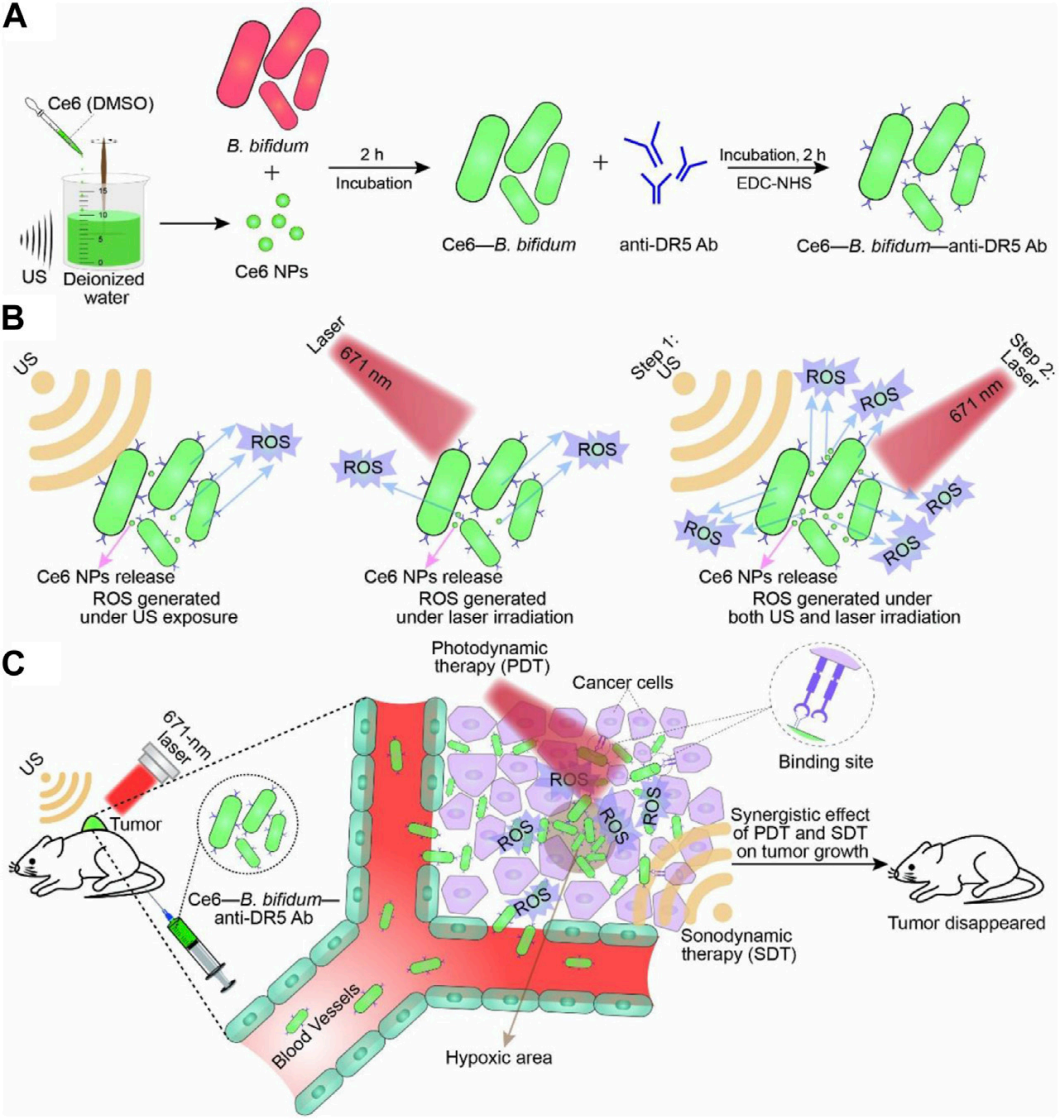
Schematic illustration of Ce6–B. bifidum–anti-DR5 Ab for tumor-targeted PDT and SDT. **(A)** Preparation of Ce6 NPs and Ce6–B. bifidum–anti-DR5 Ab. **(B)** Combination of laser and US exposure exerted a synergistic effect on ROS generation of Ce6–B. bifidum–anti-DR5 Ab. **(C)** Combined 671 nm laser and US targeted therapy. Reproduced with permission from (Li et al., 2022). ^©^ 2022 Acta Materialia Inc. Published by Elsevier Ltd. All rights reserved.

*B. bifidum* is a Gram-positive bacterium that is well-known to humans and has been used as a probiotic in the treatment of various diseases (Levit et al., 2022; Cao et al., 2023). Similar to the aforementioned bacteria, *Bifidobacteria* also have the ability to specifically target tumors and colonize and replicate within them, highlighting their potential as a delivery platform for antitumor therapy (Lu et al., 2022). Furthermore, engineered *bifidobacteria* have distinct advantages in aiding tumor therapy diagnostics. For instance, Sheethal Reghu et al. utilized *bifidobacteria* encapsulated with indocyanine green (ICG) nanoparticles to develop light-inducing functional bacteria for cancer photothermal therapy (Reghu and Miyako, 2022). In another study, Li et al. incorporated Ce6 nanoparticles into *B. bifidum* and attached an anti-death receptor five antibody to the bacterium’s surface, resulting in significant inhibition of tumor growth through laser and ultrasound irradiation (Figure 5) (Li et al., 2022). Due to its probiotic properties, *B. bifidum* exhibits good biocompatibility and significantly reduces side effects when used as a delivery platform *in vivo*, particularly for assisting photothermal and laser therapies. Apart from the synergistic effects of photothermal and laser therapies, engineered *bifidobacteria* can also be combined with immunotherapy. He et al. targeted the hypoxic environment of tumors by covalently attaching DOX-loaded CaP/SiO_2_ nanoparticles (DNPs) to *bifidobacteria*, leading to DOX chemotherapy and simultaneous induction of immunogenic cell death (ICD), which effectively suppressed metastasis of murine melanomas while controlling the primary tumors (He et al., 2023).

### 2.5 *Akkermansia muciniphila* as drug delivery vehicles

*A. muciniphila* is a typical human gut bacterium. *A. muciniphila* is negatively associated with obesity, diabetes, cardiovascular disease, and low-grade inflammation (Plovier et al., 2017; Depommier et al., 2019; Yang et al., 2019; Yoon et al., 2021). In recent years, it has been discovered that various bacteria are associated with the development and treatment of CRC (Zheng et al., 2019; Jiang et al., 2023; Kang et al., 2023). *A. muciniphila* is considered one of the most promising bacteria for suppressing tumors in CRC due to its multiple mechanisms of colorectal cancer inhibition. It inhibits colorectal tumorigenesis through three main mechanisms: i) induction of TLR2/NLRP3-mediated M1-like TAMs, ii) reprogramming of TME, and iii) purification of membrane protein-regulated CD8 T cells (Shi et al., 2020; Fan et al., 2021; Luo et al., 2021; Jiang et al., 2023). Moreover, changes in the abundance of *A. muciniphila* in the intestinal tract can help predict clinical responses to immune checkpoint inhibitors in non-small cell lung cancer and advanced melanoma (Derosa et al., 2022; Lee et al., 2022). In conclusion, *A. muciniphila* is an excellent bacterium with anti-tumor properties.

## 3 OMVs based drug delivery system

**FIGURE 6:**
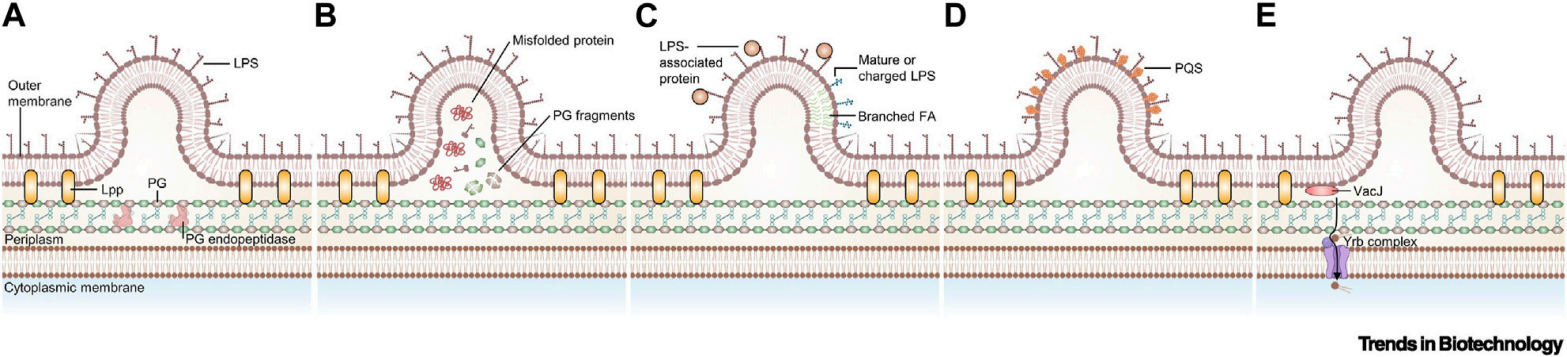
Currently proposed models for the biogenesis of outer membrane vesicles (OMVs). **(A)** Disruption of peptidoglycan–lipoprotein crosslinks (Toyofuku et al., 2017). **(B)** Accumulation of envelope component (Schwechheimer et al., 2014). **(C)** Enrichment of specific LPS in some area (Chowdhury and Jagannadham, 2013). **(D)** Insertion of the Pseudomonas quinolone signal (Tashiro et al., 2010). **(E)** Downregulation of VacJ/Yrb ABC transporter (Zingl et al., 2020). Reproduced with permission from (Xie et al., 2022). ^©^ 2022 Published by Elsevier Ltd. Abbreviations: FA, fatty acid; Lpp, lipoprotein; PG, peptidoglycan; PQS, *Pseudomonas quinolone* signal.

OMVs are active substances produced by Gram-negative bacteria in response to external stimuli (Toyofuku et al., 2019; Dell’Annunziata et al., 2021). These vesicles are mainly rich in lipopolysaccharides (LPS), phospholipids, outer membrane, and periplasmic proteins, which play a crucial role in bacterial survival and invasion (Kaparakis-Liaskos and Ferrero, 2015; Kim et al., 2015; Gerritzen et al., 2017; Wang et al., 2022). The formation of OMVs can be regulated by increasing the accumulation of relevant components on the outer membrane, altering the fluidity of the bacterial membrane, decreasing the cross-linking of peptidoglycan, and other destabilizing factors of the outer membrane (Figure 6) (Xie et al., 2022; Long et al., 2022). OMVs, when modified by tumor antigens, have been shown to induce antigenic specific humoral and cellular immunity in mice, exerting a strong inhibitory effect on tumors. Additionally, encapsulated OMVs contain virulence factors that can be delivered to host cells, stimulating bacterial-host cell interactions with intrinsic antitumor activity. This is possible due to their powerful pathogen-associated molecular patterns (PAMPs), including LPS and lipoproteins, which can be efficiently recognized and internalized by immune cells (Chen et al., 2022). Bacterial outer membrane vesicles hold promise as lipid-like bilayer nanocarriers due to their low toxicity, drug-carrying capacity, and ease of modification and industrialization (Sartorio et al., 2021). In this section, we focused on experimental advances using OMVs as a vehicle to aid in tumor therapy and diagnosis.

### 3.1 OMVs as delivery platform combined with antitumor therapy

OMVs, which are spherical NPs derived from bacteria, are highly biocompatible and ideally suited in morphology and size for carrying antitumor drug delivery in synergistic tumor diagnostics and therapy (Feng et al., 2022). Liu et al. utilized OMVs derived from E. coli to construct functionalized Fe_3_O_4_-MnO_2_ (FMO) nanoplatforms, enabling neutrophil-mediated targeted transport and photothermal enhancement of cancer immunotherapy, thereby enhancing the accumulation of FMO NPs in tumor tissues. FMO NPs undergo reactive breakdown at the tumor site to produce Mn and iron ions, induce ICD and O_2_, and regulate the hypoxic environment of the tumor. Additionally, PTT and Fe_3_O_4_ induced by MnO can directly destroy tumor cells, stimulate systemic immune responses, and promote nanoparticle enrichment of neutrophil-prepared NPs by augmenting the inflammation response at the tumor site, thus enabling immunotherapy of tumors (Liu et al., 2023). Li, Wu et al. introduced Ce6 and adriamycin together into OMVs extracted using E. coli. Ce6 acted as a photosensitizer to potentiate PDT, DOX served as a chemotherapeutic agent to kill tumor cells, and OMVs enhanced anti-tumor immunity. Together, these three components were used to eradicate triple-negative breast cancer in mice without side effects and prevent its metastasis to the lung (Li et al., 2023). Shi et al. loaded 5-FU into OMVs. They utilized OMVs modified nanocarriers mesoporous silica, combining the advantages of intestinal absorption of OMVs and the high drug loading capacity of MSNs to release 5-FU after adsorption of OMVs on the intestinal surface. This targeted delivery of 5-FU reduced the side effects of liver and spleen damage associated with conventional drug administration (Shi et al., 2020). Li et al. prepared genetically engineered OMVs inserted into the extracellular domain of programmed death protein 1 (PD1). These OMVs bind to PD-L1 on the surface of tumor cells and protect T-cells from immunosuppression caused by PD1/PD-L1, thereby enhancing the immune response (Li et al., 2020).

### 3.2 OMVs converted to a tumor vaccine

OMVs contain PAMPs that strongly stimulate the natural immune system and are often used in the preparation of tumor vaccines as immune adjuvants. In a study by Yue et al., tumor antigens were loaded along with mouse IgG fragments into the Cly A of OMVs. Subsequently, an arabinose-inducible promoter was introduced to control the expression of the fusion protein. The controlled manufacture of OMVs containing tumor antigens was achieved by orally administering modified bacteria and an expression elicitor, arabinose. It was demonstrated that this method caused significant tumor suppression in various mouse tumor models (Yue et al., 2022). In another study by Li et al., OMVs were utilized as a platform for mRNA transfer. This was achieved by modifying the RNA-binding protein L7Ae and the lysosomal escape protein Listeriolysin O (OMV-LL). The OMV-LL was then used to adsorb C/D sequence-labeled mRNA antigen (OMV-LL-mRNA), which was subsequently delivered into DCs. The mRNA antigen was presented by Listeria lysozyme O-mediated escape *in vivo*. The experimental results showed that OMV-LL-mRNA could induce long-term immune memory and protect mice from tumors after 2 months. Moreover, it caused 37.5% complete regression in a colon cancer model and significantly inhibited melanoma development (Li et al., 2022). In a study by Cheng et al., an OMV-based vaccine platform was constructed by fusing antigens with Cly A proteins to the surface of OMVs. This simplified the antigen presentation process through a “plug-and-play” approach. Multiple protein capture agents were used to modify OMVs, enabling the display of multiple different tumor antigens. This induced a synergistic anti-tumor immune response. The OMVs obtained in this manner not only eliminated melanoma lung metastases but also inhibited the growth of subcutaneous colorectal cancer (Cheng et al., 2021).

## 4 Conclusions and future prospects

The above findings suggest that *E. coli*, *Salmonella*, *LM*, *B. bifidum*, and *A. muciniphila* specifically target hypoxic tumors. Moreover, their derivative OMV significantly enhances innate immunity, serving as a potential means for drug delivery and improving the effectiveness of other therapies. Furthermore, they can be utilized as delivery platforms for tumor vaccines. Thus, adjuvant bacteria and their derivatives are highly regarded as excellent carrier platforms for antitumor drug delivery and synergistic antitumor therapy. They have become an indispensable weapon in the fight against tumors. Nevertheless, there are several issues that require attention. Firstly, it is important to consider the toxicity of drug-carrying bacteria, particularly as patients undergoing radiotherapy often have a weakened immune system. If the toxicity of the bacteria is excessive, it could overwhelm the immune system, increasing the risk of bacterial infection. To mitigate this risk and prevent adverse events, it is crucial to minimize the toxicity of the bacteria used as carriers. Secondly, to enhance the bacterial loading rate of the drug, smaller doses of bacteria can be administered to deliver a greater quantity of drugs to the targeted area, thereby reducing the dependence on high bacterial doses. Lastly, the yield of OMV is currently limited, and various issues need to be addressed to increase its production and enhance its purification. However, with the continuous advancement of modern molecular biotechnology, these challenges will be tackled, bringing renewed hope for antitumor therapy.
